# Altered stress fibers and integrin expression in the Malpighian epithelium of *Drosophila* type IV collagen mutants

**DOI:** 10.1016/j.dib.2016.03.059

**Published:** 2016-03-19

**Authors:** András A. Kiss, Nikoletta Popovics, Gábor Szabó, Katalin Csiszár, Mátyás Mink

**Affiliations:** a Department of Genetics, University of Szeged, Középfasor 52, H-6726 Szeged, Hungary; b John A. Burns School of Medicine, University of Hawaii, 1960 East West Road, Honolulu, HI 96822, USA

**Keywords:** *col4a1* mutation, Integrin misexpression, Stress fibers

## Abstract

Basement membranes (BMs) are highly specialized extracellular matrices (ECMs) that provide support and polarization cues for epithelial cells. Proper adhesion to the BM is pivotal in epithelial cell function and survival. Type IV collagens are the predominant components of all types of BMs, that form an irregular, polygonal lattice and serve as a scaffold for numerous other BM components and BM-associated cells. Mutations in the ubiquitous human BM components *COL4A1* and *COL4A2* cause a multisystem disorder involving nephropathy. Affected patients develop renal dysfunction and chronic kidney failure with or without hematuria. Mouse *Col4a1* and *Col4a2* mutants recapitulate the human symptoms. In vertebrates, excretion is accomplished by the kidneys and by the Malpighian tubules in insects, including the fruit fly *Drosophila*. Our present results with dominant, temperature-sensitive mutation of the *Drosophila col4a1* gene demonstrate altered integrin expression and amplified effects of mechanical stress on the Malpighian epithelial cytoskeleton.

**Specifications Table**

**Table:** 

Subject area	*Biology*
More specific subject area	*Genotype-phenotype relationship in Drosophila type IV collagen mutants*
Type of data	*Microscopic images, text file*
How data was acquired	*Microscope*
Data format	*Raw*
Experimental factors	*Immunohistochemistry*
Experimental features	*Confocal fluorescence pictures*
Data source location	*N.a.*
Data accessibility	*N.a.*

## Value of the data

● Mutations in the ubiquitous human basement membrane component *COL4A1* cause a systemic disease affecting the brain, muscle, blood vessels, kidneys and eyes.
● While mouse models of these mutations recapture the human phenotype, inherent limitations argue for a versatile and more easily tractable model.
● Mutations within the homologous type IV collagen gene in *Drosophila* recapitulate pathological elements of the human disease and provide suitable phenotypic markers for therapeutic evaluations.

## 1 Data

We have identified dominant, temperature-sensitive mutations within the *Drosophila* type IV collagen gene *col4a1*, the insect homologue of mammalian *COL4A1*. Similar to their mammalian counterparts, the mutations trigger a systemic phenotype, including severe myopathy, intestinal dysfunction and a robust immune response manifested by overexpression of antimicrobial peptides and excess synthesis of the oxidants hydrogen peroxide and peroxynitrite [1], [2], [3]. The Malpighian tubules, the excretory organ of insects, are functionally similar to the mammalian kidneys [4]. The tubules are freely floating within the hemocoel, the openly circulating blood-filled body cavity. We surmised that the mechanical impetus of periodic movements of the insect body that keeps the Malpighian tubules in continuous movement, may also contribute to stress-induced cytoskeletal reorganization in mutant animals.

**Fig. 1:**
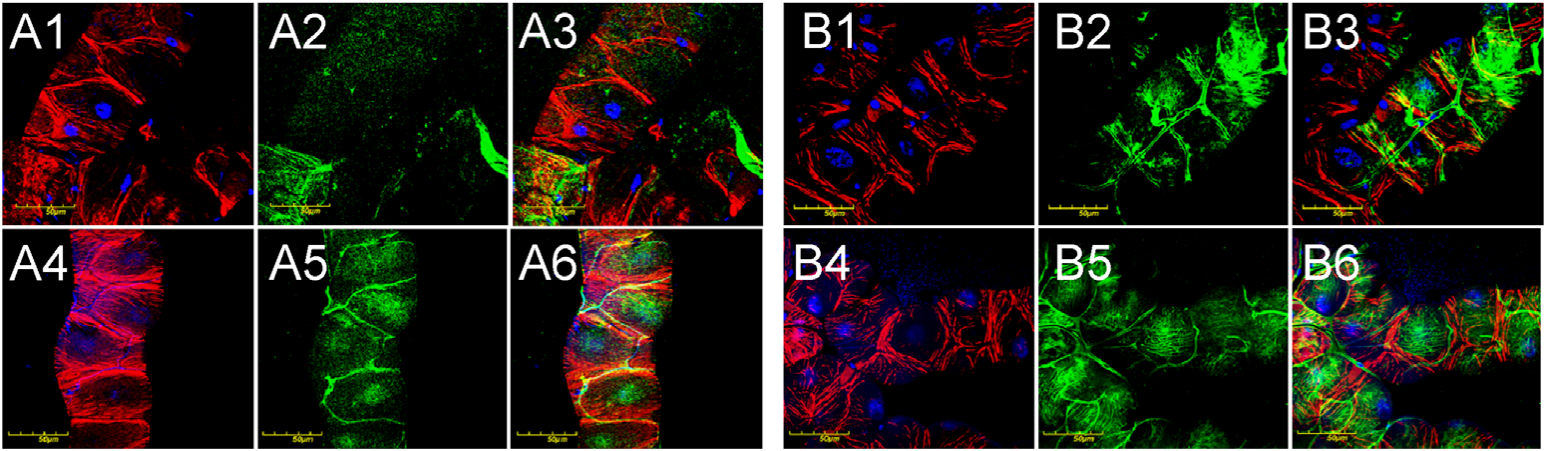
Malpighan tubules of wild-type (A1–A3 and B1–B3) and mutant (A4–A6 and B4–B6) flies kept at 20 °C for 3 and 18 days. Red: Actin, blue: DAPI-stained nuclei, green: COL4A1. A1, A4, B1, B4: Actin stained by phalloidin; A2, A5, B2, B5: COL4A1 antibody staining; A3, A6, B3, B6: Merge. Bars equal 50 μm.

**Fig. 2:**
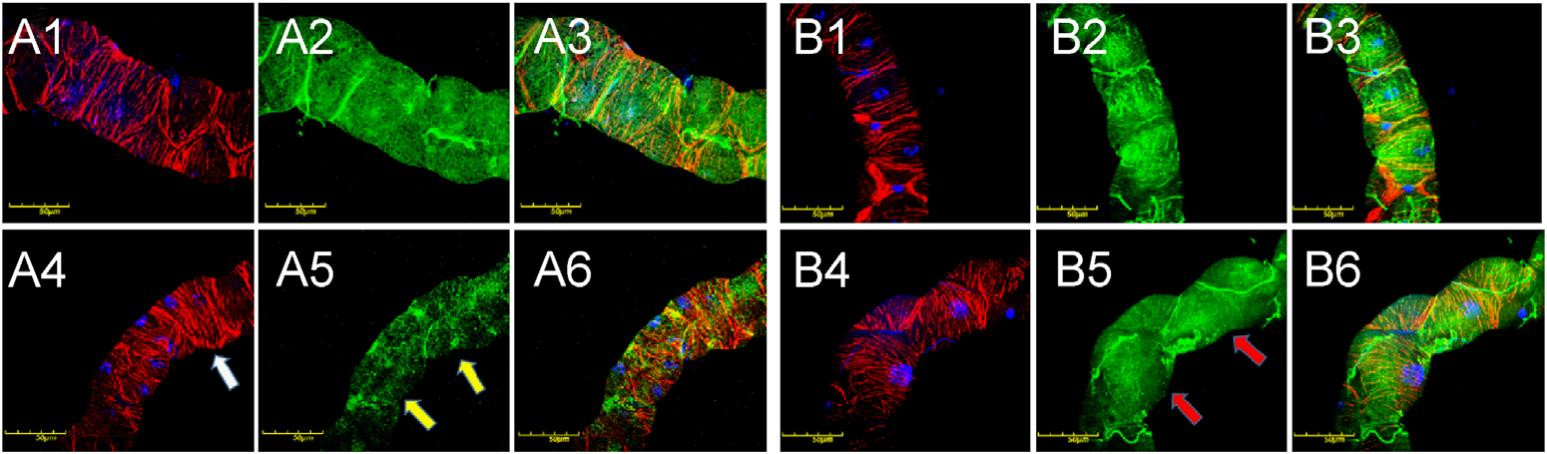
COL4A1 and stress fiber detection in Malpighan tubules of wild-type (A1–A3 and B1–B3) and mutant (A4–A6 and B4–B6) flies incubated at 29 °C for 3 and 18 days. Red: Actin, blue: DAPI-stained nuclei, green: COL4A1. A1, A4, B1, B4: Actin stained by phalloidin; A2, A5, B2, B5: COL4A1 antibody staining; A3, A6, B3, B6: Merge. Stress fibers appear in mutants following three days incubation at 29 °C (A4, white arrow) and became more abundant by day 18 (B4); mutant Malpighian tubules express less COL4A1 (A5, yellow arrows, B5, red arrows). Bars equal 50 μm.

In order to explore the phenotype of Malpighian tubules associated with mutated *Drosophila col4a1* gene, we have chosen a mutant line with the *DTS-L3* allele [1], [2], [3] as a model, together wild-type *OregonR* flies. Both lines were incubated at permissive (20 °C) and restrictive (29 °C) temperatures and evaluated following three and eighteen days of incubation. At permissive temperature, both in wild-type and mutant flies actomyosin accumulated in the cortical periphery of epithelial cells irrespective of the incubation times while COL4A1 protein staining showed regular distribution (Fig. 1). At restrictive temperature, epithelial cells of *col4a1* mutant developed actin stress fibers visible already at three days of incubation (Fig. 2, A4, white arrow) that became abundant within the cytoplasm by day 18 (Fig. 2, A5). Results were similar to cytoskeletal rearrangement reported for the proximal tubule cells in kidneys of *Col4a1*^*G498V/G498V*^ homozygous mouse mutants [5]. In the mutants, epithelial cells also expressed less COL4A1 protein both at the third (Fig. 2, A5, yellow arrows) and eighteenth day (Fig. 2, B5, red arrows) of incubation at 29 °C.

Type IV collagens of the BM bind cell-surface integrin receptors as ligands with high affinity and are parts of the cytoskeleton-extracellular matrix axis [6]. The trans-membrane integrins mediate dynamic interactions, including mechano-transduction, between the ECM/BM and the actin cytoskeleton.

To evaluate the effect of altered type IV collagen network on the BM-cytoskeletal axis in mutant animals, we examined the expression and distribution of integrin PS I alpha and PS II alpha subunits.

**Fig. 3:**
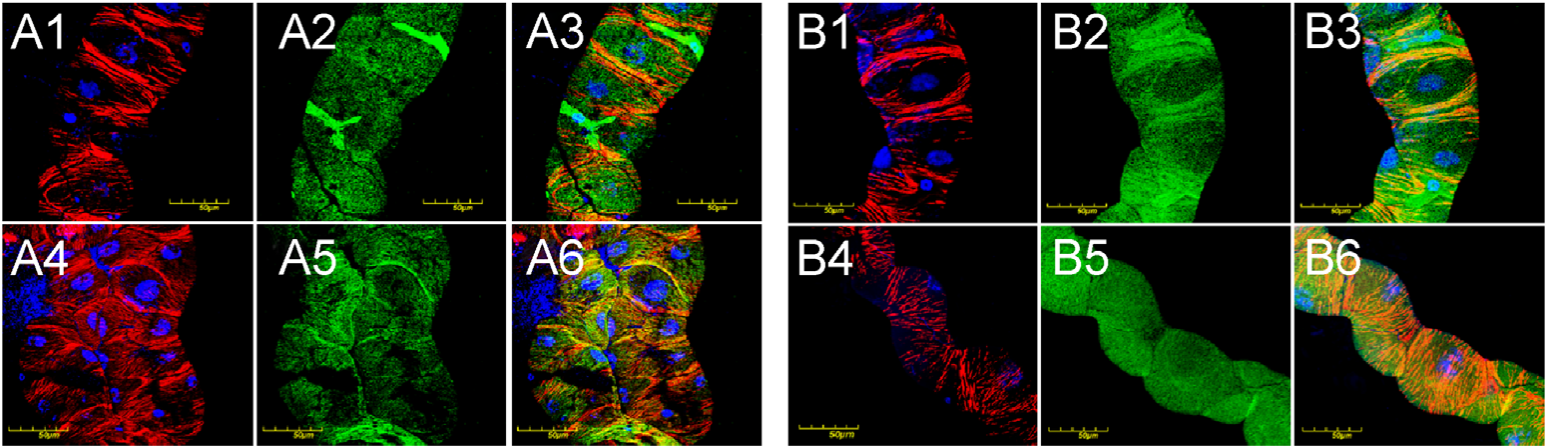
Integrin detection in Malpighan tubules of wild-type (A1–A3 and B1–B3) and mutant (A4–A6 and B4–B6) flies incubated at 20 °C for 3 and 18 days. Red: Actin, blue: DAPI-stained nuclei, green: Integrin-staining by anti-PS I alpha and PS II alpha antibodies. Bars equal 50 μm.

**Fig. 4:**
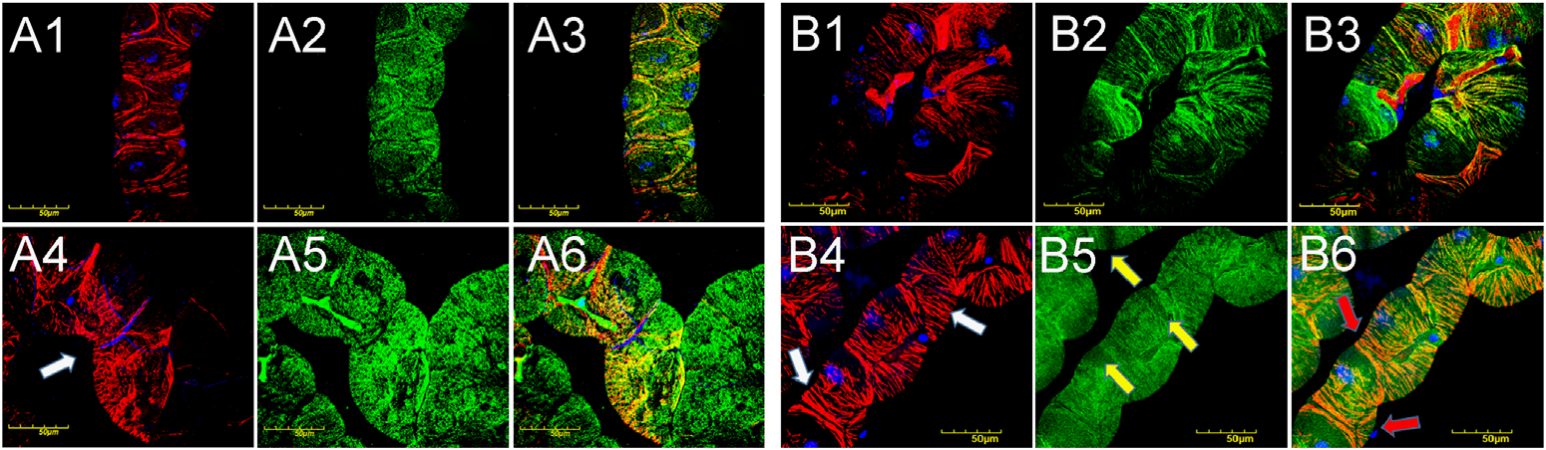
Altered integrin localization and stress fibers in Malpighan tubules of wild-type (A1–A3 and B1–B3) and mutant (A4–A6 and B4–B6) flies incubated at 29 °C for 3 and 18 days. Red: Actin, blue: DAPI-stained nuclei, green: Integrin-staining by anti-PS I alpha and PS II alpha antibodies. Stress fibers appeared following three days heat shock (A4, white arrow) and became more abundant by day 18 (B4, white arrows). Alpha integrins co-localized with actin (B3) in wild-type controls whereas in mutants, large areas showed reduced staining for integrins (B5, yellow arrow), furthermore, integrin signals did not co-localize with actin (B6, red arrows). Bars equal 50 μm.

Immunohistochemistry detected integrins as evenly distributed punctate staining at the surfaces of epithelial cells of Malpighian tubules (Fig. 3) and integrin alpha subunits also co-localized with actin staining as demonstrated by red (actin) and green (integrin) labeling in the merged photomicrographs (Fig. 3, A3, A6, B3, B6, orange) in both mutant and wild-type lines at permissive temperature irrespective of the age of the flies or length of incubation. At elevated temperature, mutant lines developed actin stress fibers by day 3 (Fig. 4, A4, white arrow) that persisted at day 18 (Fig. 4, B4, white arrows) a feature that was not observed in wild-type animals (Fig. 4, A1, B1). In mutants, integrin staining was uneven with areas of minimal expression at day 18 (Fig. 4, B5, yellow arrows). Additionally, integrin and actin connections appeared disrupted as these proteins were no longer co-localized, demonstrated by areas with isolated integrin staining (Fig. 4, B6, red arrows). In wild-type controls, integrin and actin appeared in close proximity supporting the existence of proper cytoskeleton-ECM linkage (Fig. 4, B3). The data collectively demonstrate that *col4a1* mutation affects integrin expression, causes irregular accumulation of integrins and an increase in stress fibers within the epithelial cells of Malpighian tubules due to impaired mechano-transduction. These phenotypic changes in *Drosophila* col4a1 mutant model represent cellular markers suitable for rapid and cost-effective evaluations of targeted therapeutical interventions.

## 2 Experimental design, materials and methods

Mutant and control flies were propagated on yeast-cornmeal-agar medium consisting of nipagine to prevent fungal infections. Malpighian tubules were dissected from carbon-dioxide-anesthetized wild-type and mutant flies, fixed and processed as described [1]. Mouse monoclonal antibody against *Drosophila* COL4A1 protein was generated using the peptide ATGAGSIQDS (29–38, Creative Ltd, Szeged, Hungary). To visualize integrin dimers consisting of either PS I alpha or PS II alpha subunits, an equimolar mixture of both anti-integrin monoclonal antibodies (mouse, Developmental Studies Hybridoma Bank) were utilized. Primary mouse antibodies were visualized by anti-mouse Alexa Fluor 488-conjugated secondary antibody (Invitrogen, Life Technology). Photomicrographs were taken by an Olympus Fluoview FV1000 confocal laser scanning microscope (Olympus Life Science Europa GmbH, Hamburg, Germany).

## Competing interests

The authors have declared that no competing interest exists.
