# Supplementary material for: Altered stress fibers and integrin expression in the Malpighian epithelium of Drosophila type IV collagen mutants
Source: Data Brief. 2016 Mar 19;7:868–72. doi: 10.1016/j.dib.2016.03.059 (PMC4816910; doi:10.1016/j.dib.2016.03.059)
Supplement: Supplementary file 1 — Supplementary material [file mmc1.docx]

**Competing interests**

The authors have declared that no competing interest exists.
